# Incidence of *Streptococcus* spp. and whole genome sequencing of *Streptococcus agalactiae* isolated from cow’s milk samples in Brazil

**DOI:** 10.1007/s11259-026-11393-z

**Published:** 2026-07-15

**Authors:** Rafaela Martins Morasi, Bruna Lourenço Crippa, Clarice Gebara, Hélio Langoni, Carlos Henrique Camargo, Luiz Gustavo de Matos, André Thaler Neto, Mônica Correia Gonçalves, Nathália Cristina Cirone-Silva

**Affiliations:** 1 Department of Food Sciences and Nutrition, Faculty of Food Engineering, State University of Campinas (UNICAMP), Campinas, São Paulo Brazil; 2 Food Research Center, School of Veterinary and Animal Science, Federal University of Goiás (UFG), Goiás, Goiânia Brazil; 3 Department of Animal Production and Preventive Veterinary Medicine, Faculty of Veterinary Medicine and Zootechnics, São Paulo State University (UNESP), Botucatu, São Paulo Brazil; 4 Bacteriology Center, Adolfo Lutz Institute (IAL), São Paulo, Brazil; 5 Department of Veterinary Medicine and Animal Sciences, University of Studies of Milan (UNIMI), Lodi, Lombardy, Italy; 6 Department of Animal and Food Production, University of the State of Santa Catarina (UDESC), Lages, Santa Catarina Brazil; 7 Center for Agro-food Science and Technology, Federal University of Campina Grande (UFCG), Pombal, Paraíba Brazil

**Keywords:** *Streptococcus*, Milk samples, Whole genome sequencing, Antimicrobial resistance, Virulence genes

## Abstract

This study assessed the occurrence of *Streptococcus* spp. in milk samples from cows with visible clinical mastitis signs and from cows with high (≥ 200,000 cells/mL) or low (< 200,000 cells/mL) somatic cell counts (SCC) across five Brazilian regions. Antimicrobial resistance and virulence-associated genes were investigated using phenotypic assays and whole-genome sequencing (WGS). Among 1,447 milk samples, *Streptococcus agalactiae* was the predominant species, followed by *Streptococcus uberis* and *Streptococcus dysgalactiae*, with most isolates associated with high SCC samples. Positive samples presented elevated SCC values, reinforcing the association between *Streptococcus* spp. and udder inflammation. High resistance rates to streptomycin and tetracycline were observed, and multidrug resistance was detected in one-third of the isolates. Genomic analysis of *S. agalactiae* identified two sequence types (ST-67 and ST-103), resistance genes associated with aminoglycoside, tetracycline, and macrolide resistance, and a broad repertoire of virulence-related genes. *S. agalactiae* isolates were detected exclusively in samples from Paraíba, whereas *S. uberis* was identified across all evaluated regions, suggesting distinct epidemiological patterns among *Streptococcus* species. Overall, these findings highlight the epidemiological relevance, antimicrobial resistance potential, and virulence-associated characteristics of *Streptococcus* spp. involved in bovine mastitis in Brazil, reinforcing the importance of continuous surveillance and improved mastitis control strategies in dairy herds.

**Supplementary Information:**

The online version contains supplementary material available at https://doi.org/10.1007/s11259-026-11393-z.

## Introduction

Somatic cell count (SCC) in milk is a widely recognized indicator of intramammary health due to its strong correlation with milk quality. Elevated SCC in dairy cows is primarily caused by udder infections, most often resulting from pathogenic microorganisms such as those of the *Streptococcus* genus (Moradi et al. 2020). Mastitis remains one of the most prevalent and costly diseases in dairy herds worldwide, leading to substantial economic losses due to decreased milk yield, discarded milk, treatment expenses, and increased culling rates (Ruegg et al. 2015).

*Streptococcus* spp. are among the most significant mastitis-causing pathogens in dairy cattle (Cheng and Han 2020). *Streptococcus agalactiae* is a highly contagious agent transmitted mainly during milking, as it resides in the udder and often establishes chronic infections, leading to persistently elevated SCC and reduced milk production (Cheng and Han 2020; Kibebew 2017). In contrast, *Streptococcus uberis* is generally classified as an environmental pathogen, occurring both in the host and the milking environment (Fessia and Odierno 2021; Käppeli et al. 2019), although reports of cow-to-cow transmission have added complexity to its epidemiology (Vezina et al. 2021). *Streptococcus dysgalactiae* is also an important etiological agent, with characteristics that overlap those of environmental and contagious pathogens (Zadoks et al. 2011). On the other hand, *Streptococcus equinus* is classified as an environmental pathogen and can be found as part of the intestinal and fecal flora of cattle, but it is rarely associated with bovine mastitis (Clarke et al. 2016; Kabelitz et al. 2021).

The emergence of antimicrobial-resistant *Streptococcus* isolates has been increasingly reported worldwide. Although antimicrobials remain the main therapeutic approach for mastitis, irregular or excessive use favors the selection and dissemination of resistant strains (Tian et al. 2019). The principal antimicrobial classes used in dairy herds against *Streptococcus* spp. include β-lactams, aminoglycosides, lincosamides, tetracyclines, and sulfonamides (Economou and Gousia 2015; Haenni et al. 2018; Léger et al. 2017; Redding et al. 2019). Resistance to multiple drugs can restrict therapeutic options, prolong infections, and facilitate pathogen persistence in herds.

Beyond resistance, the coexistence of antimicrobial resistance and virulence genes in *Streptococcus* isolates poses an even greater challenge for mastitis control. Virulence factors such as adhesins, invasins, capsular polysaccharides, and immune evasion proteins enable these pathogens to adapt to the bovine mammary gland, evade host defenses, and establish persistent infections (Kabelitz et al. 2021). The combination of phenotypic resistance, genetic determinants of resistance, and virulence traits can substantially increase the risk of pathogen dissemination both within and between herds.

In Brazil, studies investigating the genomic and phenotypic characteristics of *Streptococcus* spp. associated with bovine mastitis remain scarce, particularly regarding the distribution of sequence types (STs), resistance gene profiles, and virulence gene repertoires in different regions of the country. This knowledge gap limits the ability to develop targeted prevention and control strategies adapted to local epidemiological conditions.

Given this context, the present study aimed to evaluate the incidence, antimicrobial resistance, and genomic features of *Streptococcus* spp. isolated from milk samples of cows with varying SCC levels across Brazil, with a focus on *S. agalactiae* isolates from the state of Paraíba. By integrating phenotypic and genomic data, this work provides insights into the epidemiology, resistance mechanisms, and virulence potential of these pathogens in Brazilian dairy herds.

## Materials and methods

### Animal ethics

This study was approved by the Genetic Heritage Management Council of the National System of Management of Genetic Heritage and Associated Knowledge (registration number: A4784B5) and by the Ethics Chamber in the Use of Animals (CEUA) of the “Universidade Federal de Goiás” (MB number 057/21).

### Origin and acquisition of milk samples

A total of 1,447 milk samples (convenience sampling) were collected from farms in the five administrative regions of Brazil. These comprised 1,249 composite samples from all four mammary quarters of each cow and 198 individual quarter samples from clinical mastitis cases. Samples originated from cows classified into three groups according to clinical presentation and SCC values: (i) cows with visible clinical signs of mastitis; (ii) cows without clinical signs of mastitis but with high SCC (≥ 200,000 cells/mL); and (iii) cows without clinical signs and with low SCC (< 200,000 cells/mL).

In terms of herd location, collections were conducted in Santa Catarina (South), São Paulo (Southeast), Goiás (Central-West), Paraíba (Northeast), and Pará (North). Samples were obtained from 17 farms: six in Paraíba, four in Santa Catarina, three each in Pará and Goiás, and one in São Paulo. The distribution of samples by state and sample type is shown in Table 1.

**Table 1:** Relationship between the number of milk samples per state of Brazil and sample type (clinical mastitis, high SCC, and low SCC)

Sample type	São Paulo *n* (%)	Pará *n* (%)	Paraíba *n* (%)	Santa Catarina *n* (%)	Goiás *n* (%)	Total *n* (%)
Visible signs of clinical mastitis	122 (34.5%)	4 (1.3%)	30 (10.8%)	36 (14.0%)	6 (2.3%)	**198 (13.7%)**
High SCC without visible clinical signs	98 (27.8%)	69 (23.1%)	166 (59.5%)	118 (45.7%)	78 (30.2%)	**529 (36.6%)**
Low SCC	133 (37.7%)	226 (75.6%)	83 (29.7%)	104 (40.3%)	174 (67.5%)	**720 (49.7%)**
Total	**353**	**299**	**279**	**258**	**258**	**1**,**447**

Milk samples were collected in sterile bottles after antisepsis of the teat orifice with 3.0% iodinated alcohol. During sampling, no animals were undergoing antimicrobial treatment. Clinical mastitis was diagnosed by observing local and/or systemic changes in the milk, confirmed by the strip cup test.

Milk samples were obtained through a multicenter collaborative research project involving researchers from different Brazilian regions. Farms were selected by convenience sampling based on sample availability, the proximity of each researcher’s workplace, and the participation of collaborating research groups associated with the project.

The samples were transported to the Department of Food Science and Nutrition (UNICAMP, Campinas, São Paulo, Brazil) under refrigeration (4 to 8 °C) in isothermal boxes containing recyclable ice via air or land transportation.

### Determination of SCC values by flow cytometry technique

SCC analysis was performed using the flow cytometry technique on Fossomatic 5000basic^®^ equipment following the procedures recommended by the International Standard ISO 13366-2 (ISO 2006). Analyses were conducted at the Milk Quality Laboratory of the Federal University of Goiás (UFG), Goiânia, Brazil. According to Normative Instruction No. 76 (Brasil 2018), milk samples with SCC ≥ 200,000 cells/mL were classified as high SCC, and those with SCC < 200,000 cells/mL as low SCC.

### Isolation and identification of *Streptococcus* spp.

The identification of *Streptococcus* spp. followed the methodology described by Brito et al. (1999). A 10 µL aliquot of each milk sample was plated on 5.0% sheep blood agar (Oxoid, Basingstoke, UK) and incubated at 37 °C for 24–48 h. Colonies were evaluated for morphology, size, pigmentation, and hemolysis. Colonies with characteristics consistent with *Streptococcus* spp. were subjected to Gram staining and catalase testing to confirm genus identity (Reyher et al. 2012; Tomazi et al. 2018). Species-level identification was performed using Matrix Associated Laser Desorption-Ionization – Time of Flight (MALDI-TOF) (Almeida et al. 2024; Barcelos et al. 2019) at the Milk Quality Research Laboratory, Department of Nutrition and Animal Production, Faculty of Veterinary Medicine and Animal Science, University of São Paulo (USP, Pirassununga, São Paulo, Brazil).

Species identification was performed using the MBT Biotyper Compass v.4.1.100 database according to the manufacturer’s scoring criteria, with MALDI-TOF scores ≥ 2.0 considered reliable for species-level identification. In this study, only isolates meeting this threshold were assigned to the species level based on the proprietary reference library. While this approach provides high confidence in species identification, reliance on a single reference database may reduce detection sensitivity and potentially underestimate the diversity of species associated with bovine mastitis.

### Disk diffusion test for antimicrobial susceptibility of *Streptococcus* spp.

Each isolate was inoculated into Brain Heart Infusion (BHI; Oxoid) broth and incubated at 35 °C for 18–24 h. The bacterial suspension was then standardized to 0.5 McFarland in 0.85% NaCl solution and plated on Mueller–Hinton agar (Oxoid) supplemented with 5.0% sheep blood. Antimicrobial discs were applied firmly to the agar surface, maintaining adequate spacing between them. Plates were incubated at 35 °C for 18–24 h (EUCAST 2022; Matuschek et al. 2014), and inhibition zones were measured using a digital caliper.

The antimicrobials tested against *Streptococcus* spp. and their respective concentrations were: amoxicillin-clavulanic acid (AMC, 30 µg), clindamycin (CLI, 2 µg), streptomycin (EST, 10 µg), neomycin (NEO, 30 µg), cephalothin (CFL, 30 µg), oxacillin (OXA, 1 µg), tetracycline (TET, 30 µg), and sulfamethoxazole-trimethoprim (SUT, 25 µg). The antimicrobial panel was selected based on the clinical relevance of these compounds in veterinary medicine, their use in bovine mastitis treatment, and their frequent inclusion in antimicrobial susceptibility studies involving mastitis-associated *Streptococcus* spp.

Groups A, B, C, and G *Streptococcus* isolates were tested according to the guidelines of the European Committee on Antimicrobial Susceptibility Testing (EUCAST 2022). For pathogen-antimicrobial combinations without interpretative criteria in EUCAST, breakpoints established by Bauer et al. (1966), were adopted for EST, NEO, and CFL, as described by Petrovski et al. (2015).

### DNA extraction

Based on the disk diffusion test results, nine multidrug-resistant (MDR) isolates of *Streptococcus agalactiae*, defined as resistant to three or more antimicrobial classes, were selected for whole genome sequencing (WGS). To ensure culture purity, each isolate was inoculated into Brain Heart Infusion (BHI) broth and incubated at 37 °C for 24 h, followed by plating on CHROMagar™ StrepB (PlastLabor, São Paulo, SP, Brazil). After incubation, a single colony was re-isolated on BHI agar plates and incubated under the same conditions.

For DNA extraction, one colony from each pure culture was suspended in 100 µL of Milli-Q water in a 1.5 mL microtube and centrifuged at 12,000 rpm for 5 min. The supernatant was discarded, leaving only the pellet for extraction. Genomic DNA was extracted using the DNeasy^®^ PowerFood^®^ Microbial Kit (Qiagen, Valencia, CA, USA), following the manufacturer’s instructions. DNA concentration and purity were assessed using a NanoDrop Lite spectrophotometer (Thermo Fisher Scientific, Waltham, MA, USA).

### Whole genome sequencing of *Streptococcus agalactiae*

The sequencing library was prepared using the QIAseq FX DNA Library Core Kit (24 samples) (Qiagen, Hilden, Germany), following the manufacturer’s instructions. Library quality was assessed with the High Sensitivity D5000 ScreenTape Assay on a TapeStation system (Agilent Technologies, Santa Clara, CA, USA) and quantified by quantitative PCR using the QIAseq Library Quant Assay Kit (Qiagen, Hilden, Germany),

Sequencing was performed at the High-Performance Multi-User Sequencing Facility of the Brazilian Biorenewables National Laboratory (LNBR), part of the Brazilian Centre for Research in Energy and Materials (CNPEM, Campinas, São Paulo, Brazil), using an Illumina MiSeq platform.

Sequence alignment, assembly, antimicrobial resistance and virulence gene detection were conducted using Kraken2 (version 2.1.3), Unicycler (version 0.5.1), MLST, ABRicate, ABRicate Summary, Prokka (version 1.14.6), and Roary (version 3.13.0) software tools (Atxaerandio-landa et al. 2022) implemented via the Galaxy Europe (version 25.0.3) platform (The Galaxy Community 2022).

Phylogenetic analysis was performed using the Bacterial and Viral Bioinformatics Resource Center (BV-BRC) platform (https://www.bv-brc.org/) with the codon tree method. This method selects PGFams (Protein Families) and aligns proteins and coding sequences from single-copy genes using RAxML. Model parameters were automatically optimized by RAxML to generate a maximum likelihood (ML) tree, applying 25 rate categories per site, 100 bootstrap replicates, and the GAMMA model. The phylogenetic tree was visualized and edited using iTOL Version 7.1.1 (https://itol.embl.de/). The sequence project has been deposited at GenBank under the BioProject accession PRJNA1305436.

### Statistical data

Descriptive analyses were performed for the distribution of *Streptococcus* spp., sample classification groups (visible clinical mastitis signs, high SCC, and low SCC), antimicrobial resistance profiles, and multidrug resistance patterns. Categorical variables were expressed as absolute frequencies and percentages. SCC measurements were summarized using median values due to the non-normal distribution of the data. Given the descriptive and exploratory nature of the study, no inferential statistical analyses were performed.

## Results

### Isolation, identification, and distribution of Streptococcus spp

**Fig. 1:**
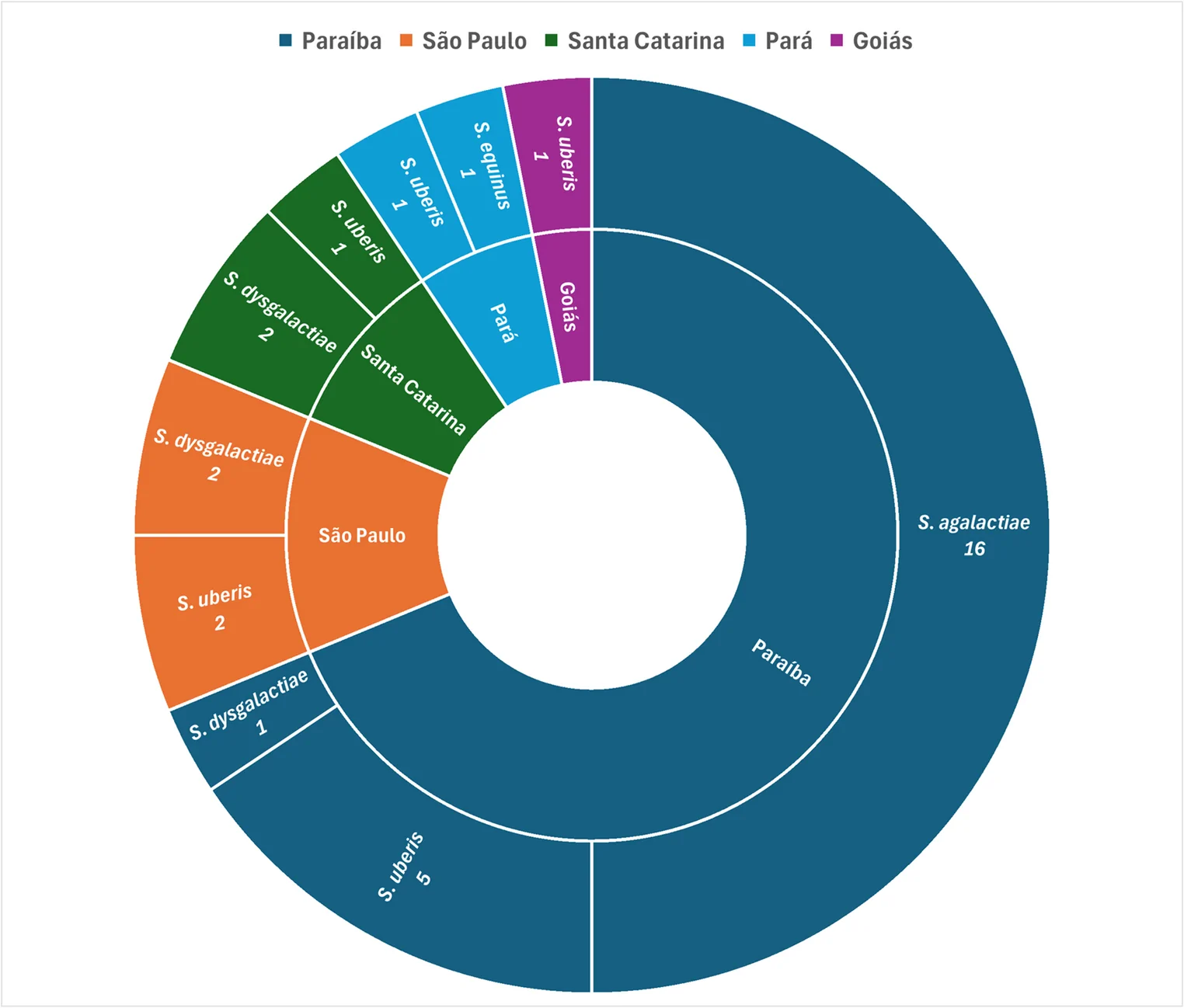
Distribution of *Streptococcus* spp. isolates identified in milk samples according to the Brazilian states included in this study

From the 1,447 milk samples, 32 were positive for *Streptococcus* spp., including 50.0% (*n* = 16) *S. agalactiae*, 31.3% (*n* = 10) *S. uberis*, 15.6% (*n* = 5) *S. dysgalactiae*, and 3.1% (*n* = 1) *S. equinus*. Regarding Brazilian states, Paraíba presented the highest number of *Streptococcus* isolates: 72.7% (*n* = 16) of *S. agalactiae*, 22.7% (*n* = 5) of *S. uberis*, and 4.6% (*n* = 1) of *S. dysgalactiae*, totaling 68.7% (*n* = 22) of all isolates. Moreover, at least one *S. uberis* isolate was identified in each state included in the study (Fig. 1).

### Association between *Streptococcus* spp. isolates and SCC in bovine milk

**Fig. 2:**
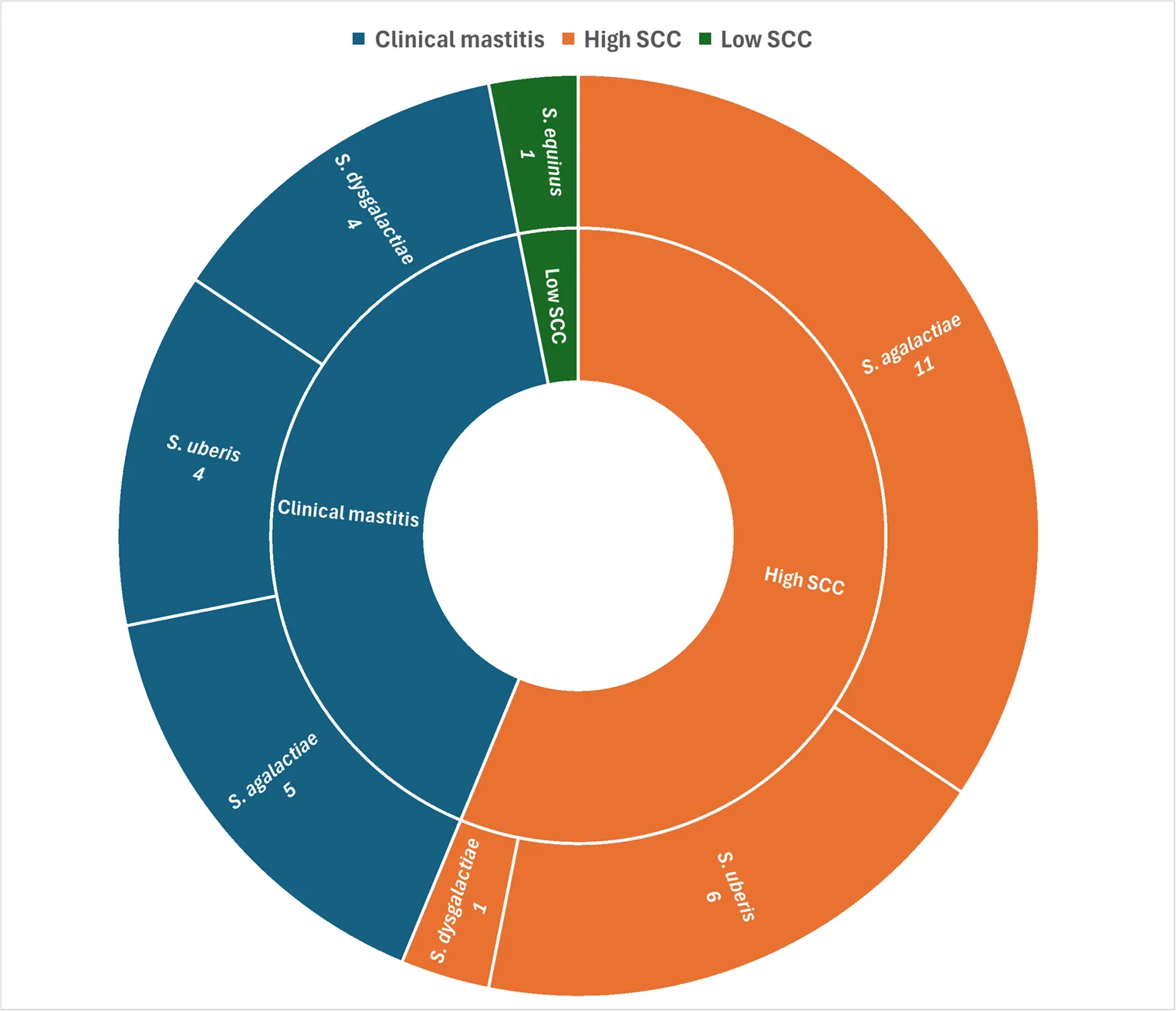
Distribution of *Streptococcus* spp. isolated from milk samples associated with visible clinical mastitis signs, high SCC, and low SCC

Of the 32 *Streptococcus* spp. isolates, 56.3% (*n* = 18) were identified from milk samples with high SCC, 40.6% (*n* = 13) from visible clinical mastitis cases, and 3.1% (*n* = 1) from low SCC samples, classified as *S. equinus* (Fig. 2). The median SCC of milk samples positive for *Streptococcus* spp. was 1,696,000 cells/mL.

### Antimicrobial resistance profiles and multidrug resistance in *Streptococcus* spp. isolates

*Streptococcus* spp. isolates exhibited the highest resistance rates to streptomycin (EST), with 59.4% (*n* = 19) resistant strains, followed by tetracycline (TET) with 50.0% (*n* = 16), neomycin (NEO) with 37.5% (*n* = 12), oxacillin (OXA) and sulfamethoxazole-trimethoprim (SUT) each with 25.0% (*n* = 8), and clindamycin (CLI) with 18.8% (*n* = 6). No resistance was detected to cephalothin (CFL) or amoxicillin-clavulanate (AMC).

**Fig. 3:**
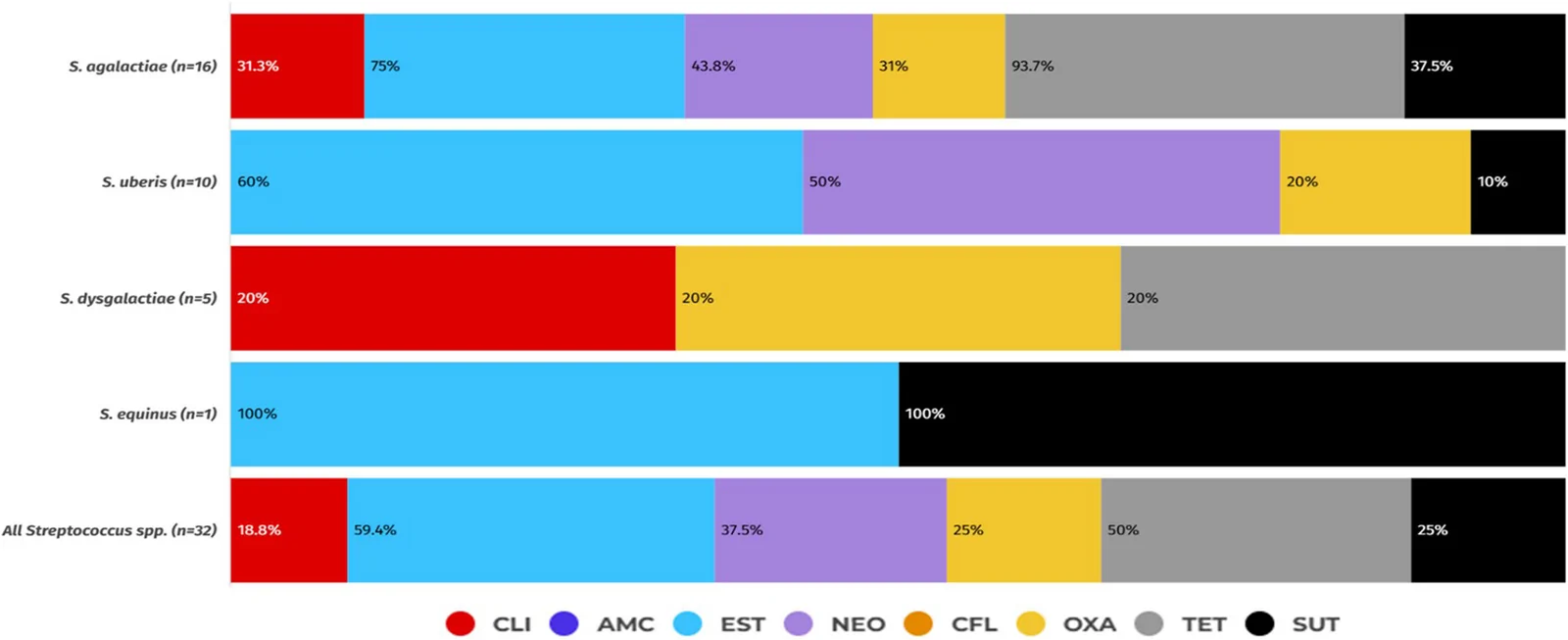
Antimicrobial resistance profiles of *Streptococcus* spp. isolates. CLI: clindamycin (class of lincosamides); AMC: amoxicillin with clavulanic acid (class of β-lactams); EST: streptomycin (class of aminoglycosides); NEO: neomycin (class of aminoglycosides); CFL: cephalothin (class of cephalosporin); OXA: oxacillin (class of β-lactams); TET: tetracycline (class of tetracyclines); SUT: sulfamethoxazole-trimethoprim (class of sulfonamides); S: sensitive; I: intermediate; R: resistant

At the species level, *S. agalactiae* showed the highest resistance rates, with 93.7% (*n* = 15) resistant to TET and 75.0% (*n* = 12) to EST. Most *S. uberis* and *S. dysgalactiae* isolates were susceptible to the tested antimicrobials; however, 60.0% (*n* = 6) of the 10 *S. uberis* isolates were resistant to EST, and 20.0% (*n* = 1) of the five *S. dysgalactiae* isolates exhibited simultaneous resistance to CLI, OXA, and TET. The single *S. equinus* isolate was resistant only to EST and SUT (Fig. 3).

**Fig. 4:**
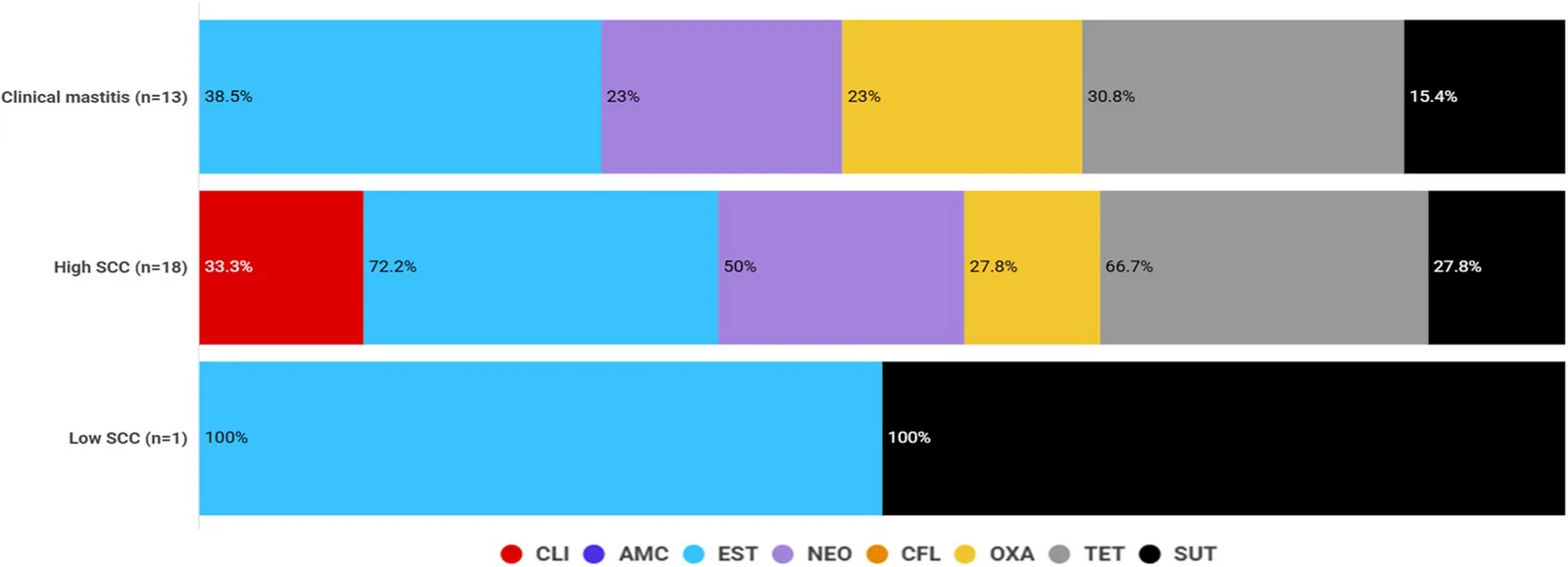
Antimicrobial resistance profiles of *Streptococcus* spp. isolates according to sample type. CLI: clindamycin (class of lincosamides); AMC: amoxicillin with clavulanic acid (class of β-lactams); EST: streptomycin (class of aminoglycosides); NEO: neomycin (class of aminoglycosides); CFL: cephalothin (class of cephalosporin); OXA: oxacillin (class of β-lactams); TET: tetracycline (class of tetracyclines); SUT: sulfazotrim (class of sulfonamides); S: sensitive; I: intermediate; R: resistant

Regarding udder health status, the single isolate from a low SCC sample was susceptible to all tested antimicrobials except EST and SUT. In samples with high SCC and visible clinical mastitis signs, EST showed the highest resistance rates, with 72.2% (*n* = 13) and 38.5% (*n* = 5) of isolates resistant, respectively (Fig. 4).

Among the 32 isolates, 34.4% (*n* = 11) were classified as multidrug-resistant (MDR), defined as resistance to at least three antimicrobial classes. Of these, 81.8% (*n* = 9) were *S. agalactiae*, and 9.1% (*n* = 1) each were *S. uberis* and *S. dysgalactiae*. Considering the sample type, 72.8% (*n* = 8) of MDR isolates were from high SCC milk and 27.2% (*n* = 3) from visible clinical mastitis cases. Of the high SCC group, 75.0% (*n* = 6) were *S. agalactiae*, 12.5% (*n* = 1) *S. uberis*, and 12.5% (*n* = 1) *S. dysgalactiae*. All MDR isolates from visible clinical mastitis milk samples (100.0%; *n* = 3) were *S. agalactiae*. The state of Paraíba accounted for the highest number of MDR isolates, with 90.9% (*n* = 10), of which 72.8% (*n* = 8) were resistant to three antimicrobial classes and 27.2% (*n* = 3) to four classes.

### Whole genome sequencing of *Streptococcus agalactiae*

A total of nine *S. agalactiae* isolates from bovine milk samples collected in the state of Paraíba were subjected to whole genome sequencing. Genome sizes ranged from 1,975,829 to 2,153,068 bp, with the number of contigs per genome varying between 18 and 42 (Supplementary Table 1).

**Fig. 5:**
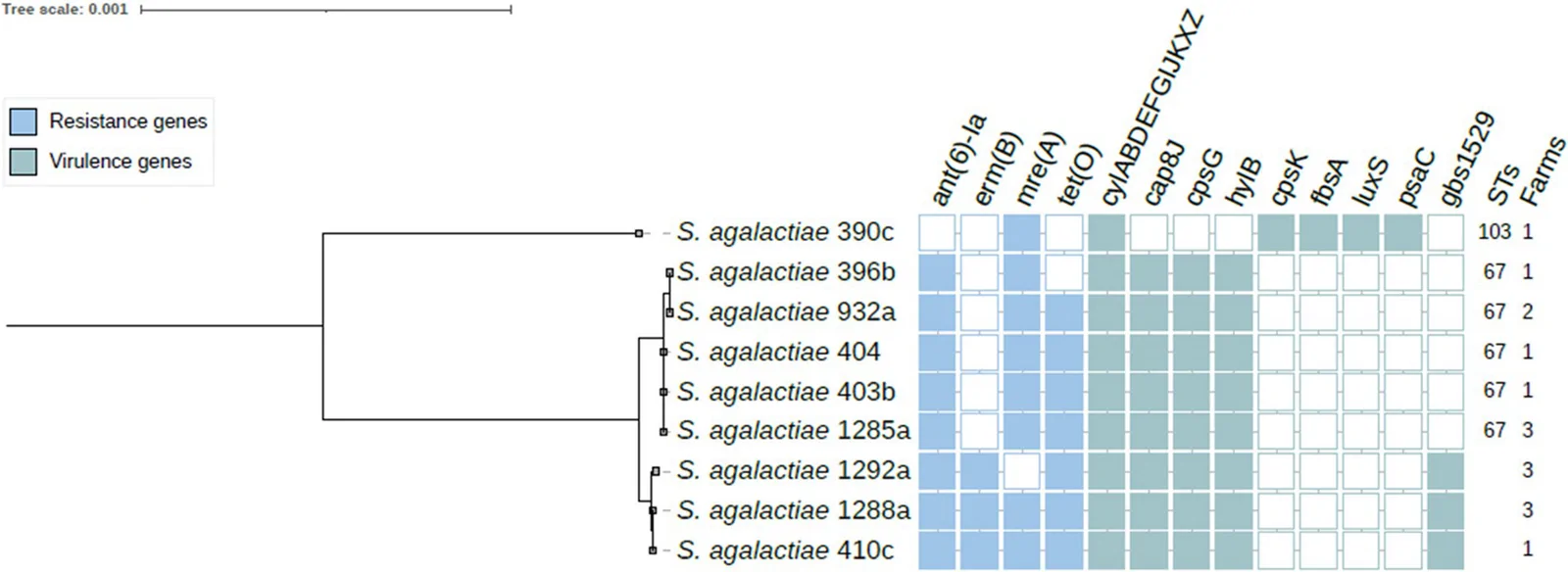
Phylogenetic tree of *Streptococcus agalactiae* isolates from bovine milk, showing sequence type (ST), farm origin, and presence of antimicrobial resistance and virulence genes

Multilocus Sequence Typing (MLST) identified two predominant sequence types (STs): ST-67 in five isolates (55.6%) and ST-103 in one isolate (11.1%). Three isolates (33.3%) could not be assigned to a known ST (UN). The phylogenetic tree revealed the formation of three distinct clades, consistent with ST classification (Fig. 5).

When considering ST distribution by farm, the sequenced isolates originated from three (50.0%) of the six farms sampled in Paraíba (designated Farm 1, Farm 2, and Farm 3 to maintain confidentiality). Farm 1 harbored the greatest diversity, with isolates belonging to both identified STs and the UN group (Fig. 3).

Genomic analysis identified antimicrobial resistance genes consistent with the phenotypic resistance patterns observed in the disk diffusion tests. The aminoglycoside resistance gene *ant(6)-Ia* was detected in isolates showing phenotypic resistance to streptomycin (EST), while macrolide resistance genes *erm(B***)** and *mef(A)* were found in isolates resistant to clindamycin (CLI). Similarly, the tetracycline resistance gene *tet(O***)** was identified in isolates phenotypically resistant to tetracycline (TET).

Regarding virulence-associated genes, genes related to capsular polysaccharide synthesis (*cps* operon), surface proteins (*lmb* and *fbs*A), hyaluronidase (*hyl*B), and manganese transport (*psa*C) were identified among the sequenced isolates.

#### Identification of antimicrobial resistance genes

Considering the four antimicrobial resistance genes, a relationship between ST and resistance gene profile was observed, except for sample 1292a (ST unidentified), which lacked *mre(A).* Notably, only samples 410c and 1288a harbored all four resistance genes simultaneously (Fig. 3).

All phenotypically resistant isolates identified in the disk diffusion test carried the corresponding resistance genes in their genome. However, discrepancies were found in two cases: isolate 390c was phenotypically resistant to tetracycline (TET) and streptomycin (EST) but did not harbor the corresponding resistance genes, and isolate 1285a showed phenotypic resistance to clindamycin (CLI) without carrying *erm(B*).

Macrolide-class antimicrobials were not included in the phenotypic testing, precluding correlation analysis for *mre(A)* and *erm(B)* activity. In contrast, β-lactam, cephalosporin, and sulfonamide classes were tested phenotypically, but no corresponding resistance genes were detected.

#### Identification of virulence genes

WGS identified a total of 77 virulence genes, subsequently grouped according to their functional roles (Supplementary Table 2). Four distinct distribution patterns were observed (Table 2).

**Table 2:** Distribution patterns of virulence genes in *Streptococcus agalactiae* isolates, according to sequence type and functional category

Functional category	Virulence genes detected
Core virulence genes (present in all isolates)	*acpC*,* bca*,* carB*,* ccpA*,* cfa/cfb*,* ciaR*,* clpP*,* cpsA*,* cpsB*,* cpsC*,* cpsD*,* cpsE*,* cpsF*,* cpsL*,* cpsY*,* csrR*,* csrS*,* cydA*,* cylA*,* cylB*,* cylD*,* cylE*,* cylF*,* cylG*,* cylI*,* cylJ*,* cylK*,* cylX*,* cylZ*,* fba*,* gbs1402*,* glnA*,* guaA*,* hasC*,* lepA*,* leuS*,* lgt*,* murF*,* neuA*,* neuB*,* neuC*,* neuD*,* pbp1A*,* pepC*,* pepN*,* pepX*,* purB*,* purH*,* purL*,* purN*,* rpoE*,* SAK_0517*,* scrB*,* sodA*,* SP_0095*,* SP_0121*,* SP_0320*,* SP_0494*,* SP_0829*,* SP_0856*,* SP_0943*,* SP_1396*,* SP_1398*,* SP_1399*,* SP_1544*,* SP_1847*,* SP_1970*,* vicK*
Absent in ST-103	*cap8J*,* cpsG*,* hylB*
Exclusive to ST-103	*cpsK*,* fbsA*,* luxS*,* psaC*
Exclusive to ST-unidentified isolates	*gbs1529*

Overall, isolates 390c, 410c, 1288a, and 1292a harbored 73 virulence genes each (94.8%), while isolates 396b, 403b, 404, 932a, and 1285a carried 72 genes each (93.5%).

## Discussion

This study investigated the occurrence, antimicrobial resistance, and genomic characteristics of *Streptococcus* spp. isolated from bovine milk samples collected in different Brazilian regions. Among the identified species, *S. agalactiae* was the most frequent, followed by *S. uberis* and *S. dysgalactiae*. Most isolates were recovered from high SCC samples and samples with visible clinical mastitis signs, reinforcing the association between *Streptococcus* spp. and intramammary inflammation.

*S. uberis* was identified in all evaluated states, consistent with its widespread distribution in dairy environments and its importance as both an environmental and opportunistic mastitis pathogen (Tomazi et al. 2019; Wente et al. 2019). In contrast, all *S. agalactiae* isolates originated from Paraíba within the present sampling design. As a contagious pathogen, *S. agalactiae* is commonly associated with persistent intramammary infections, elevated SCC, and recurrent mastitis cases (Leelahapongsathon et al. 2016; Rossi et al. 2018). The elevated median SCC observed among positive samples in this study agrees with previous reports describing the impact of *Streptococcus* infections on udder inflammation and milk quality (Djabri et al. 2002; Malinowski et al. 2006).

The detection of *S. equinus* in a low SCC sample deserves attention. *Streptococcus equinus* is classified as an environmental pathogen and is commonly found as part of the intestinal and fecal flora of cattle, but it is only rarely associated with bovine mastitis and remains poorly characterized regarding its role in intramammary infections (Clarke et al. 2016; Verdier-metz et al., 2012; Kabelitz et al. 2021). Therefore, its detection in a low SCC sample may reflect transient contamination or limited inflammatory response.

Antimicrobial susceptibility testing demonstrated high resistance rates to streptomycin and tetracycline, particularly among *S. agalactiae* isolates. Similar resistance patterns have been reported in previous studies involving bovine mastitis-associated *Streptococcus* spp. (Minst et al. 2012; Rato et al. 2013; Haenni et al. 2018). In contrast, β-lactams and cephalosporins remained highly effective against the isolates analyzed, supporting their continued relevance for mastitis treatment. The presence of multidrug-resistant isolates, especially among *S. agalactiae*, highlights the importance of continuous surveillance and prudent antimicrobial use in dairy herds. The antimicrobial panel was selected based on the clinical relevance of these compounds in veterinary medicine, their use in bovine mastitis treatment, and their frequent inclusion in antimicrobial susceptibility studies involving mastitis-associated *Streptococcus* spp. Because veterinary-specific interpretive criteria were unavailable for certain antimicrobial agents or *Streptococcus* species, susceptibility results were interpreted using EUCAST guidelines complemented by published references. Although this approach enabled the evaluation of all antimicrobial agents included in the study, the use of heterogeneous breakpoint systems may have influenced the classification of resistance for some antimicrobials and may limit direct comparisons among antimicrobial agents and with studies adopting different interpretive criteria. Therefore, the resistance rates reported here should be interpreted in light of the reference standards available for each antimicrobial.

WGS of *S. agalactiae* isolates identified resistance genes associated with aminoglycoside, tetracycline, macrolide, and lincosamide resistance. In most cases, the detected genes corresponded to the phenotypic resistance profiles observed in disk diffusion assays. However, some isolates displayed phenotypic resistance without detection of the corresponding genes, suggesting the possible involvement of alternative resistance mechanisms, such as chromosomal mutations or regulatory changes (Carattoli 2001; Gao et al. 2012).

Phylogenetic analysis identified the sequence types ST-67 and ST-103 among the sequenced isolates. Both STs have previously been associated with bovine mastitis isolates in different countries, including Brazil, Norway, China, and Colombia (Carvalho-Castro et al. 2017; Jørgensen et al. 2016; Cobo-Ángel et al. 2018, 2019). The detection of different sequence types among isolates from the same region demonstrates genomic variability within the analyzed dataset. However, broader epidemiological interpretations should be made with caution due to the limited number of sequenced isolates and their geographic concentration in a single Brazilian state.

Genomic analysis also identified a broad repertoire of virulence-associated genes related to capsular polysaccharide synthesis, adhesion, immune evasion, stress response, and tissue invasion. Important virulence determinants included genes from the *cps* locus, *cyl* operon, *hyl*B, and *cfa*/*cfb*, which have previously been associated with persistence and pathogenicity in the bovine mammary gland (Amaral et al. 2022; Lin et al. 2021). Although most virulence genes were highly conserved among isolates, some lineage-specific differences were observed, particularly in the ST-103 isolate and in isolates without identified sequence type, indicating genomic variability among the analyzed strains.

Overall, the integration of phenotypic and genomic analyses provided relevant insights into the epidemiology, antimicrobial resistance, and virulence-associated characteristics of *Streptococcus* spp. involved in bovine mastitis in Brazil. These findings reinforce the importance of continuous surveillance, appropriate antimicrobial stewardship, and effective mastitis control strategies in dairy herds.

## Conclusion

This study demonstrated the occurrence of *Streptococcus* spp. in bovine milk samples from Brazilian dairy herds, including cows with visible signs of clinical mastitis and varying SCC levels. *S. agalactiae* was the predominant species identified and was mainly recovered from samples with high SCC values, whereas *S. uberis* was detected in all evaluated states, indicating its broad geographic distribution. In the present sample set, *S. agalactiae* isolates were detected exclusively in Paraíba.

Antimicrobial susceptibility testing revealed high resistance rates to streptomycin and tetracycline. In addition, multidrug-resistant *S. agalactiae* isolates carried resistance genes and virulence-associated factors related to adhesion, immune evasion, persistence, and host invasion, with ST-67 and ST-103 identified among the sequenced isolates. These findings indicate the presence of antimicrobial resistance and virulence determinants in the analysed isolates and highlight the importance of monitoring their distribution in dairy herds.

Overall, the integration of phenotypic and genomic analyses provided important insights into the occurrence, antimicrobial resistance, and virulence potential of *Streptococcus* spp. isolated from bovine milk in Brazil. These findings highlight the need for continuous surveillance and the implementation of integrated mastitis control strategies, including the prevention of cow-to-cow transmission through proper milking equipment maintenance, post-milking teat disinfection, and dry cow management, as well as the reduction of environmental exposure by improving hygiene and minimizing contact with contaminated bedding and manure.

Our findings reinforce the importance of continued epidemiological surveillance and further studies involving larger and more representative sample sets to improve the understanding of *Streptococcus* spp. distribution and antimicrobial resistance patterns in Brazilian dairy herds.

## Supplementary Information

Below is the link to the electronic supplementary material.

Supplementary Material 1 (DOCX 28.1 KB)

## Data Availability

No datasets were generated or analysed during the current study.

## References

[CR1] Almeida JM, De, Maffei JT, Gebara C, Minafra C, Toledo-Silva B, Gonçalves MC et al (2024) Exploring probiotic potential and antimicrobial properties of lactic acid bacteria from cow’s milk. Appl Food Res 4:100461. 10.1016/j.afres.2024.100461

[CR2] Amaral V, Thiago R, Kato RB, Soares SDC, Matiuzzi M (2022) Bacteriocin Producing *Streptococcus agalactiae* Strains Isolated from Bovine Mastitis in Brazil. Microorganisms 10(588):1–26. 10.3390/microorganisms10030588 PMC8953382 35336163

[CR3] Atxaerandio-landa A, Arrieta-gisasola A, Laorden L, Bikandi J, Garaizar J, Martinez-Malaxetxebarria I et al (2022) A practical bioinformatics workflow for routine analysis of bacterial WGS data. Microorganisms 10:1–13. 10.3390/microorganisms10122364 PMC9781918 36557617

[CR4] Barcelos MM, Martins L, Grenfell RC, Juliano L, Anderson KL, Santos MV et al (2019) Comparison of standard and on-plate extraction protocols for identification of mastitis-causing bacteria by MALDI-TOF MS. Brazilian J Microbiol 50:849–857. 10.1007/s42770-019-00110-5 PMC6863300 31256351

[CR5] Bauer AW, Kirby MD, Sherris WMMMD (1966) Antibiotic susceptibility testing by a standardized single disk method. Am J Clin Pathol 45(4):493–496 5325707

[CR6] Brasil (2018) Ministério da Agricultura, Pecuária e Abastecimento. Instrução Normativa No76, de 26 de novembro de 2018. Accessed on 16.04.2026. https://www.in.gov.br/materia/-/asset_publisher/Kujrw0TZC2Mb/content/id/52750137/do1-2018-11-30-instrucao-normativa-n-76-de-26-de-novembro-de-2018-52749894IN 76

[CR7] Brito MAVP, Brito JRF, Ribeiro MT, Veiga VMO (1999) Padrão de infecção intramamária em rebanhos leiteiros: exame de todos os quartos mamários das vacas em lactação. Arq Bras Med Veterinária e Zootec 51(2):129–135

[CR8] Burnside K, Lembo A, Harrell MI, Gurney M, Xue L, Connelly JE et al (2011) The serine / threonine phosphatase Stp1 mediates post transcriptional regulation of hemolysin, autolysis and virulence of Group B Streptococcus. Am Soc Biochem Mol Biol 11. 10.1074/jbc.M111.313486 PMC3243546 22081606

[CR9] Carattoli A (2001) Review article Importance of integrons in the diffusion of resistance. Vet Res 32:243–259 11432416 10.1051/vetres:2001122

[CR10] Carvalho-Castro GA, Silva JR, Paiva LV, Custódio DAC, Moreira RO, Mian GF et al (2017) Molecular epidemiology of *Streptococcus agalactiae* isolated from mastitis in Brazilian dairy herds. Brazilian J Microbiol 48(3):551–559. 10.1016/j.bjm.2017.02.004 PMC5498452 28256391

[CR11] Cheng WN, Han SG (2020) Bovine mastitis: risk factors, therapeutic strategies, and alternative treatments - A review. Asian-Australasian J Anim Sci 33(11):1699–1713. https://pubmed.ncbi.nlm.nih.gov/32777908/10.5713/ajas.20.0156 PMC7649072 32777908

[CR12] Chimalapati S, Cohen JM, Camberlein E, Macdonald N, Durmort C, Vernet T et al (2012) Effects of deletion of the *Streptococcus pneumoniae* lipoprotein diacylglyceryl transferase gene *lgt* on ABC transporter function and on growth *in vivo*. PLoS One. 10.1371/journal.pone.0041393 22911788 PMC3404074

[CR13] Clarke LL, Fathke RL, Sanchez S, Stanton JB (2016) *Streptococcus bovis/S. equinus* complex septicemia in a group of calves following intramuscular vaccination. J Vet Diagn Investig 28(4):423–428 27216720 10.1177/1040638716648364

[CR14] Cobo-Ángel C, Jaramillo-Jaramillo AS, Lasso-Rojas LM, Aguilar-Marin SB, Sanchez J, Rodrigues-Lecompte JC et al (2018) *Streptococcus agalactiae* is not always an obligate intramammary pathogen: Molecular epidemiology of GBS from milk, feces and environment in Colombian dairy herds. PLoS ONE 13(12):1–14. 10.1371/journal.pone.0208990 PMC6287850 30532177

[CR15] Cobo-Ángel CG, Jaramillo-Jaramillo AS, Palacio-Aguilera M, Jurado-Vargas L, Calvo-Villegas EA, Ospina-Loaiza DA et al (2019) Potential group B *Streptococcus* interspecies transmission between cattle and people in Colombian dairy farms. Sci Rep 9(14025):1–9. 10.1038/s41598-019-50225-w 31575879 PMC6773701

[CR16] Djabri B, Bareille N, Beaudeau F, Seegers H (2002) Original article Quarter milk somatic cell count in infected dairy cows: a meta-analysis. Vet Res 33:335–357. 10.1051/vetres:2002021 12199362

[CR18] Economou V, Gousia P (2015) Agriculture and food animals as a source of antimicrobial-resistant bacteria. Infect Drug Resist 8:49–61. 10.2147/IDR.S55778 25878509 PMC4388096

[CR19] EUCAST. The European Committee on Antimicrobial Susceptibility Testing. Breakpoint tables for interpretation of MICs and zone diameters. Version 12.0 (2022) 2022;1–110. Accessed on 16.04.2026. http://www.eucast.org

[CR21] Fessia AS, Odierno LM (2021) Potential factors involved in the early pathogenesis of *Streptococcus uberis* mastitis: a review. Folia Microbiol (Praha). 10.1007/s12223-021-00879-9 34085166

[CR22] Gao J, Yu F-Q, Luo L-P, He J-Z, Hou R-G, Zhang H-Q et al (2012) Antibiotic resistance of *Streptococcus agalactiae* from cows with mastitis. Vet J 194(3):423–424. 10.1016/j.tvjl.2012.04.020 22627045

[CR23] Haenni M, Lupo A, Madec J-Y (2018) Antimicrobial resistance in *Streptococcus* spp. Microbiol Spectr 6(2):ARBA0008-2017. 10.1128/microbiolspec.ARBA-0008-2017 PMC11633561 29600772

[CR26] ISO/IDF 2006. international dairy federation.ISO 13366-2 / IDF 148-2 - Milk - Enumeration of somatic cells – Part. 2: Guidance on the operation of fluoro-opto-electronic counters. ISO (2006) ;2006. https://www.iso.org/standard/40260.html

[CR28] Jørgensen HJ, Nordstoga AB, Sviland S, Zadoks RN, Sølverød L, Kvitle B et al (2016) *Streptococcus agalactiae* in the environment of bovine dairy herds – rewriting the textbooks? Vet Microbiol 184:64–72. 10.1016/j.vetmic.2015.12.014 26854346

[CR29] Kabelitz T, Aubry E, van Vorst K, Amon T, Fulde M (2021) The role of *Streptococcus* spp. in bovine mastitis. Microorganisms 9(7):1–21. 10.3390/microorganisms9071497 PMC8305581 34361932

[CR30] Käppeli N, Morach M, Zurfluh K, Corti S, Nüesch-inderbinen M, Stephan R (2019) Sequence Types and Antimicrobial Resistance Profiles of *Streptococcus uberis* Isolated From Bovine Mastitis. Front Vet Sci 6(July):1–7. 10.3389/fvets.2019.00234 31380400 PMC6646518

[CR31] Kibebew K (2017) Bovine Mastitis: A Review of Causes and Epidemiological Point of View. J Biol Agric Healthc 7(2):1–14

[CR33] Leelahapongsathon K, Schukken YH, Pinyopummintr T, Suriyasathaporn W (2016) Comparison of transmission dynamics between *Streptococcus uberis* and *Streptococcus agalactiae* intramammary infections. J Dairy Sci 99(2):1418–26. 10.3168/jds.2015-9950 26686709

[CR34] Léger DF, Newby NC, Reid-smith R, Anderson N, Pearl DL, Lissemore KD et al (2017) Estimated antimicrobial dispensing frequency and preferences for lactating cow therapy by Ontario dairy veterinarians. Cont Vet J 58(1):26–34 PMC5157734 28042151

[CR35] Lin L, Huang X, Yang H, He Y, He X, Huang J et al (2021) Molecular epidemiology, antimicrobial activity, and virulence gene clustering of *Streptococcus agalactiae* isolated from dairy cattle with mastitis in China. J Dairy Sci 104(4):4893–4903. 10.3168/jds.2020-19139 33551160

[CR37] Malinowski E, Lassa H, Ossowska AKŁ, Markiewicz H, Kaczmarowski MŁ, Smulski S (2006) Relationship between mastitis agents and somatic cell count in foremilk samples. Bull Vet Inst Pulawy 50(25):349–352

[CR38] Matuschek E, Brown DFJ, Kahlmeter G (2014) Development of the EUCAST disk diffusion antimicrobial susceptibility testing method and its implementation in routine microbiology laboratories. Clin Microbiol Infect 20(4):255–266. 10.1111/1469-0691.12373 24131428

[CR41] Minst K, Märtlbauer E, Miller T, Meyer C (2012) Short communication: *Streptococcus* species isolated from mastitis milk samples in Germany and their resistance to antimicrobial agents. J Dairy Sci 95(12):6957–62. 10.3168/jds.2012-5852 22999286

[CR42] Moradi M, Omer AK, Razavi R, Valipour S, Guimarães JT (2020) The relationship between milk somatic cell count and cheese production, quality and safety: a review. Int Dairy J 104884. 10.1016/j.idairyj.2020.104884

[CR46] Petrovski KR, Grinberg A, Williamson NB, Abdalla ME, Parkinson TJ, Tucker IG et al (2015) Susceptibility to antimicrobials of mastitis-causing Staphylococcus aureus, Streptococcus uberis and Str. dysgalactiae from New Zealand and the USA as assessed by the disk diffusion test. Aust Vet J 93:227–233. 10.1111/avj.12340 26113347

[CR48] Rato MG, Bexiga R, Florindo C, Cavaco LM, Vilela CL, Santos-Sanches I (2013) Antimicrobial resistance and molecular epidemiology of streptococci from bovine mastitis. Vet Microbiol 161(3–4):286–294. 10.1016/j.vetmic.2012.07.043 22964008

[CR49] Redding LE, Bender J, Baker L (2019) Quantification of antibiotic use on dairy farms in Pennsylvania. J Dairy Sci 102(2):1494–1507. 10.3168/jds.2018-15224 30594359

[CR51] Reyher KK, Haine D, Dohoo IR, Revie CW (2012) Examining the effect of intramammary infections with minor mastitis pathogens on the acquisition of new intramammary infections with major mastitis pathogens — A systematic review and meta-analysis. J Dairy Sci 95(11):6483–6502. 10.3168/jds.2012-5594 22981582

[CR52] Rossi RS, Amarante AF, Correia LBN, Guerra ST, Nobrega DB, Latosinski GS et al (2018) Diagnostic accuracy of Somaticell, California Mastitis Test, and microbiological examination of composite milk to detect *Streptococcus agalactiae* intramammary infections. J Dairy Sci 101(11):10220–10229. 10.3168/jds.2018-14753 30146291

[CR53] Ruegg PL, Oliveira L, Jin W, Okwumabua O (2015) Phenotypic antimicrobial susceptibility and occurrence of selected resistance genes in gram-positive mastitis pathogens isolated from Wisconsin dairy cows. J Dairy Sci 98(7):4521–4534. 10.3168/jds.2014-9137 25912858

[CR59] The Galaxy Community (2022) The Galaxy platform for accessible, reproducible and collaborative biomedical analyses: 2022 update. Nucleic Acids Res 50(April):345–51 10.1093/nar/gkac247 PMC9252830 35446428

[CR60] Tian XY, Zheng N, Han RW, Ho H, Wang J, Wang YT et al (2019) Antimicrobial resistance and virulence genes of *Streptococcus* isolated from dairy cows with mastitis in China. Microb Pathog 13:33–39. 10.1016/j.micpath.2019.03.035 30940606

[CR61] Tomazi T, Ferreira GC, Orsi AM, Gonçalves JL, Ospina PA, Nydam DV et al (2018) Association of herd-level risk factors and incidence rate of clinical mastitis in 20 Brazilian dairy herds. Prev Vet Med 16:9–18. 10.1016/j.prevetmed.2018.10.007 30466663

[CR62] Tomazi T, Freu G, Alves BG, Francisco A, Filho DS, Heinemann MB et al (2019) Genotyping and antimicrobial resistance of Streptococcus uberis isolated from bovine clinical mastitis. PLoS ONE 14(10):1–15. 10.1371/journal.pone.0223719 PMC6805098 31639136

[CR63] Turinsky AJ, Moir-blais TR, Grundy FJ, Henkin TM (2000) *Bacillus subtilis* ccpA Gene Mutants Specifically Defective in Activation of Acetoin Biosynthesis. J Bacteriol 182(19):5611–5614. 10.1128/jb.182.19.5611-5614.2000 10986270 PMC111010

[CR64] Verdier-metz I, Gagne G, Bornes S, Monsallier F, Veisseire P, Delbès-paus C (2012) Cow Teat Skin, a Potential Source of Diverse Microbial Populations. Appl Environ Microbiol 78(2):326–333 22081572 10.1128/AEM.06229-11 PMC3255753

[CR65] Vezina B, Al H, Ramay HR, Soust M, Moore RJ, Olchowy TWJ et al (2021) Sequence characterization and novel insights into bovine mastitis – associated *Streptococcus uberis* in dairy herds. Sci Rep [Internet]. 10.1038/s41598-021-82357-3. 1–16 33542314 PMC7862697

[CR66] Wente N, Klocke D, Paduch J, Zhang Y, Seeth M, Reinecke F et al (2019) Associations between *Streptococcus uberis* strains from the animal environment and clinical bovine mastitis cases. J Dairy Sci 102(10):9360–9. 10.3168/jds.2019-16669 31421887

[CR67] Zadoks RN, Middleton JR, McDougall S, Katholm J, Schukken YH (2011) Molecular Epidemiology of Mastitis Pathogens of Dairy Cattle and Comparative Relevance to Humans. J Mammary Gland Biol Neoplasia 16:357–372. 10.1007/s10911-011-9236-y 21968538 PMC3208832

