# Supplementary material for: Incidence of Streptococcus spp. and whole genome sequencing of Streptococcus agalactiae isolated from cow’s milk samples in Brazil
Source: Vet Res Commun. 2026 Jul 15;50(5):457. doi: 10.1007/s11259-026-11393-z (PMC13373013; doi:10.1007/s11259-026-11393-z)
Supplement: Supplementary file 1 — Supplementary Material 1 (DOCX 28.1 KB) [file 11259_2026_11393_MOESM1_ESM.docx]

**Suplemmentary Table 1.** Metadata, sequencing statistics, and quality control metrics of the sequenced *Streptococcus agalactiae* isolates.

| Isolate | Genome size (Mb) | Nº of contigs | N50 values (kb) | GC percent (%) | Genome coverage | Quality analysis  (CheckM) | Assembly coverage (%) |
| --- | --- | --- | --- | --- | --- | --- | --- |
| 390c | 2 | 15 | 221 | 35.5 | 861x | Genome completeness was 98.98% with contamination below 1% | 89.2 |
| 396b | 2.1 | 89 | 44.7 | 35.5 | 916x | Genome completeness was 97.33% with contamination below 1% | 87.2 |
| 403b | 2.1 | 98 | 40.6 | 35.5 | 909x | Genome completeness was 97.33% with contamination below 1% | 87.62 |
| 404 | 2.1 | 93 | 41.8 | 35.5 | 912x | Genome completeness was 97.82% with contamination below 1% | 87.67 |
| 410c | 2.1 | 97 | 42 | 35.5 | 936x | Genome completeness was 98.31% with contamination below 1% | 86.29 |
| 932a | 2.1 | 89 | 45.7 | 35.5 | 917x | Genome completeness was 97.33% with contamination below 1% | 87.21 |
| 1285a | 2.1 | 95 | 41.1 | 35.5 | 913x | Genome completeness was 97.82% with contamination below 1% | 87.65 |
| 1288a | 2.1 | 131 | 32.6 | 35.5 | 939x | Genome completeness was 98.31% with contamination below 1% | 86.18 |
| 1292a | 2.1 | 111 | 39.7 | 35.5 | 938x | Genome completeness was 97.74% with contamination below 1% | 86.2 |

**Supplementary Table 2.** Virulence genes identified in the Whole Genome Sequencing of *Streptococcus agalactiae* isolates, grouped according to similar functions.

| Genes | Functions | References |  |
| --- | --- | --- | --- |
| *cyl*XDG-*acp*-*cyl*ZABEFIJK; *cps*A,B,C,D,E,F,K,L; *neu*A,B,C,D; *sod*A | Evasion of the host immune system | (Burnside et al., 2011; Kabelitz et al., 2021; Amaral et al., 2022) |  |
| *Fbs*A | Pathogen’s invasion | (Rajagopal, 2009) |  |
| *car*B*; ccp*A*; clp*P*; gln*A*;* *leu*S*; pep*X*; psa*C*; pur;* SP_1970; SP_1544; SP_0856; SP_1847; SP_0494; SP_1396, SP_1398; SP_1399; SP_0829; SP_0095 | Regulation of bacterial metabolism | (Turinsky et al., 2000; Hava & Camilli, 2002; Kwon et al., 2003; Mccluskey et al., 2004; Samen et al., 2007; Luque-almagro et al., 2011; Dwyer et al., 2015; Sullivan et al., 2021) |  |
| *bca*; *csr*R,S | Host cell adhesion | (Park et al., 2012) |  |
| *Lux*S | Quorum sensing | (Reinoso, 2017) |  |
| *pep*C | Promotes changes in host’s enzymes | (Meyer, 1995) |  |
| *clp*P; *cyd*A*; gua*A*; lgt*; *lep*A *rpo*E; *scr*B; *vic*K | Bacterial growth, survival and adaptive advantages | (Hiratsuka et al., 1998; Seepersaud et al., 2006; Senadheera et al., 2009; Pech et al., 2011; Chimalapati et al., 2012) |  |
| *gbs*1402 | Resistance to multiple antimicrobials | (Fernandez et al., 2010) |  |
| *mur*F; *pep*N; *cap*8J | Development of a protective bacterial cell wall | (Sau et al., 1997; Nganje et al., 2019) |  |
| *pbpl*A; *has*C | Preserves the pathogen against phagocytosis | (Jones et al., 2003) |  |
| *gbs*1529; SP_0121; SP_0320; SP_0943 | Unknown functions | (Hava & Camilli, 2002; Samen et al., 2007) | |
